# Assessing the Consequences of External Reference Pricing for Global Access to Medicines and Innovation: Economic Analysis and Policy Implications

**DOI:** 10.3389/fphar.2022.815029

**Published:** 2022-04-06

**Authors:** András Incze, Zoltán Kaló, Jaime Espín, Éva Kiss, Sophia Kessabi, Louis P. Garrison

**Affiliations:** ^1^ Department of Healthcare Management, Baden-Wuerttemberg Cooperative State University, Loerrach, Germany; ^2^ Akceso Advisors AG, Basel, Switzerland; ^3^ Center for Health Technology Assessment, Semmelweis University/Syreon Research Institute, Budapest, Hungary; ^4^ Syreon Research Institute, Budapest, Hungary; ^5^ Andalusian School of Public Health, Granada, Spain; ^6^ The Comparative Health Outcomes, Policy, and Economics (CHOICE) Institute Department of Pharmacy, University of Washington, Seattle, WA, United States

**Keywords:** External Reference Pricing, Patient Access, Pharmaceutical Innovation, Price Regulation, U.S., Worldwide

## Abstract

**Background:** External reference pricing (ERP) is used to set pharmaceutical prices to improve affordability, but its application may have negative consequences on patient access—thus, equity—across countries and on global innovation. With the United States contemplating ERP, negative effects could be magnified. Our aim: identify and quantify some major consequences of ERP. *Research design, methods*: Besides relying on databases and ERP modelling, we developed a heart failure case study. 4-step approach: 1) review ERP policies; 2) establish worldwide “price corridor”; 3) quantify patient access and health outcomes impact by ERP; 4) estimate ERP impact on innovation.

**Results:** Our ERP referencing analysis highlights its perverse effects especially in lower-income countries. As counterstrategies to protect their revenues, manufacturers often implement tight list price corridors or launch avoidance/delays. Consequences include suboptimal patient access—hence, worse outcomes—illustrated by our case study: 500,000 + QALYs health loss. Additionally, the ensuing revenue reduction would likely cause innovation loss by one additional medicine that would have benefitted future patients.

**Conclusion:** This research provides key insights on potential unintentional consequences of medicine price setting by ERP worldwide and under a new proposal for the United States. Our results can inform stakeholder discussions to improve patient access to innovative medicines globally.

## 1 Introduction

### 1.1 Background

External reference pricing (ERP), also referred to as “external price referencing” or “international reference pricing”, is defined by the World Health Organization as “the practice of using the price(s) of a pharmaceutical product in one or several countries in order to derive a benchmark or reference price for the purposes of setting or negotiating the price of the product in a given country” (Espín et al., 2011).

Guidelines recommend applying ERP in combination with other pharmaceutical pricing instruments; however, in many countries it is currently the main or only criterion (Vogler et al., 2015; Habl et al., 2018; WHO, 2020).

ERP is used in numerous countries worldwide (OECD, 2008) [OECD Health Policy Studies, 2008], and its use is growing despite some predictions to the contrary (Persson and Jönsson, 2016). Of 31 European countries, only two are not applying it: furthermore, seven of them apply an algorithm that takes the lowest country price in their basket of comparator countries as a reference (Rémuzat et al., 2015). The price setting mechanisms for pharmaceuticals in the United States are based on complex negotiations amongst the private and public players. Price setting in the United States has, however, not been relying on ERP thus far. Following analyses over the past years—e.g., by The PEW Charitable Trusts (2017) (The Pew Charitable Trusts, 2017)—in 2019/2020, and again in 2021, the United States government, however, proposed to introduce ERP for part of the Medicare program, with one method being yet another version of the lowest country price comparison (Ways and Means Committee, 2019; Congressional Budget Offi, 2021). Most countries applying ERP do so both for determining or negotiating the launch price of the medicine and for post-launch periodic review and price revisions. Comparator country baskets are determined by each country individually, often including countries with very different healthcare systems, wealth status, epidemic, GDP, and public health situation compared to their own.

The recent WHO guideline on country pharmaceutical pricing policies concludes, nonetheless: “On balance, existing evidence suggests that external reference pricing is likely to deliver more desirable than undesirable effects, as indicated by: some (un-appraised) evidence on price reduction at least in the short term (albeit limited in the quantity and quality of evidence); a lack of robust evidence attributing undesirable effects to external reference pricing, including launch delays or product withdrawals in lower-income countries by the manufacturers to avoid prices being referenced; and wide adoption or consideration of external reference pricing as one part of the overall pricing policy” (WHO, 2020).

Given the widespread use of ERP around the globe with each country defining its own set of reference countries, calculation algorithms, and price review intervals, the result is a complex set of pricing outcomes that are sometimes unexpected or even unintended. With no general coordination mechanism for ERP across the countries, many countries cross-reference each other, thereby creating numerous circular references (Toumi et al., 2014). An added complication is the presence of “soft rules”: individual country practices may deviate from their stated regulations. Furthermore, independent within-country policies not directly related to ERP rules—such as general price cuts in difficult economic periods, product-specific price cuts, or internal price referencing with other similar drugs—can produce unintended indirect effects (Gronde et al., 2017) that can ripple through the global ERP “network.” Exchange rate fluctuations are yet a further element that can have such unpredictable impacts. For example, over the year 2020 alone, the EUR-GBP exchange rate has fluctuated by over 13%. Furthermore, even if an ERP-based price review for a given drug in a given country were to produce a higher reference price than the current one, in most countries the price increase would not be granted, either because the respective country uses ERP only for setting the initial price, or because there is explicit limitation on price increases. As a result, trends in the list prices of patented medicines are typically only downward—with the United States as the most notable exception.

Some theoretically justified approaches to “value-based differential pricing”, such as the one proposed by Danzon et al. (2015) (Danzon et al., 2015), would not require countries to reference each other’s prices. But currently as a consequence of all the above practices, pharmaceutical prices are mainly determined globally through an interdependent network that is a complex interactive and nonlinear system. This system produces both static and dynamic effects that are often difficult to foresee from the isolated perspective and perception of individual countries. As a recent conference editorial put it: “many policy-makers and payers around the globe have become aware of the weakness of existing pharmaceutical policies such as EPR” (Abstracts from the 4th In, 2019), and the potential introduction of it in the United States can be considered counterproductive to patient access, too (Fuhr et al., 2019).

ERP has the tendency to promote a narrower range—or tighter “corridor”—of prices across countries than may be desirable under the goal of “optimal global differential pricing” (Danzon and Towse, 2003). In a dynamic global marketplace, a narrow price corridor can have inconsistent, inefficient, and inequitable consequences for countries with different economic status. Across countries, ERP raises the bottom of the pricing corridor, thereby using more of the limited healthcare budgets of lower-income countries, while lowering the top of the corridor, thus allowing the “free-riding” of higher-income countries on the willingness of pharmaceutical companies to adjust drug prices to local purchasing power in lower-income countries (Csanádi et al., 2018; Holtorf et al., 2019). Untenable price levels in lower income countries (Young et al., 2017) can also result in public cost containment policies including both price and volume control measures (Elek et al., 2017; Inotai et al., 2020) while higher income countries seem to provide relatively equal access to essential treatments (Morgan et al., 2017).

In response to ERP policies and practices by the individual countries, profit-maximizing pharmaceutical companies will follow economic incentives (Lakdawalla, 2018) within the constraints and opportunities of this global ERP network to reduce or prevent the international price erosion. For example, they will apply “launch sequence” planning to achieve a higher price corridor and slower price erosion (Kanavos et al., 2020). They will avoid launching in smaller countries with lower price potential, not to jeopardize the price in larger ones (Bauer, 2017). Or they may introduce “gross-to-net” (GTN) strategies to maintain a higher list price while matching willingness and ability to pay of individual countries by providing confidential discounts (Vogler et al., 2020). Or they may withdraw a product to protect their pricing corridor. And/or in some countries they may accept volume control measures to maintain higher prices while limiting the total volume of use and sales, via 1) price-volume agreements, or 2) limiting reimbursement to only a subgroup of eligible patients, to specific prescribing centers, or on a “named-patient basis access” (Löblová et al., 2019).

All of these actions can limit patient access to innovative pharmaceuticals—especially in countries with poor economic status, which can exacerbate the health gap or inequities among populations and countries with different income levels (Kaló et al., 2013).

### 1.2 Objectives

The goal of this analysis is to identify and to quantify some of the major consequences—intended and unintended—of ERP. To do so, we characterize and model the pricing practices and related incentives in the global ERP network. We assess empirically how individual countries’ choice of ERP instruments and the resulting effects are related to their national income levels as a proxy for their “ability to pay/reimburse.” Also, we review the consequences—overt and covert—of ERP for pharmaceutical prices and patient access. Using a case study of heart failure patients, we quantify the impact of access limitations to an innovative medicine on population health. We also describe the potential long-run impact on innovation, as a reflection of dynamic (in)efficiency. Finally, we discuss the likely impact of the current proposals to implement ERP in the United States, including the effects in the United States and worldwide.

## 2 Materials and Methods

The information for these analyses originated from a combination of desk research, Akceso Advisors’ global pharmaceutical policies database and global pricing database, and a drug sales report from IQVIA, one of the largest Healthcare Data Science Companies. The geographic scope of the analyses included in total 37 countries worldwide with highly developed healthcare systems: the 27 EU countries, Australia, Brazil, Canada, Japan, New Zealand, Norway, South Korea, Switzerland, the United Kingdom and United States. These are all key countries referenced in ERP-based worldwide “system”—or perhaps “network” is a more apt label. Most use ERP to reference other countries for price benchmarking or they are in the reference basket of the other countries.

Our analysis explored four ERP-related aspects or perspectives of interest: 1) the worldwide ERP network, 2) country prices, 3) patient access, and 4) innovation impact.

### 2.1 Worldwide ERP Network

To assess the impact through the worldwide ERP network, a macroeconomic perspective (i.e., focusing on country GDP), was taken to summarize the extent to which individual countries rely on referencing prices in the countries of similar economic status or not. Nominal GDP per capita (GDP/capita) was used as a proxy for the ability to pay of a country. The most recent (2017) complete set of GDP/capita information was used from WHO. The ERP rules and algorithms were drawn from Akceso Advisors’ global pharmaceutical policies database. For the United States, two of the officially published potential algorithms were applied for comparison (Ways and Means Committee, 2019; Congressional Budget Offi, 2021). The country ERP rules and algorithms were applied to the country GDP/capita values, to calculate the country-specific GDP/capita of the ERP basket (“basket GDP/capita”) for each country. The basket GDP/capita of each country was compared to its GDP/capita to see whether a country was using an ERP methodology to compare “like with like” or not.

### 2.2 Country Prices: The Price Corridor

To establish the “worldwide price corridor” among 37 countries, the list of all centrally-registered products in Europe during the first half of 2015 was taken as the basis for all of the 37 countries in scope. The rationale for basing this on year 2015 data was that—given typical delays in some of the countries for pricing and reimbursement negotiations—this would allow follow-up to 2020. Thus, within these 5 years, it can be expected that all countries would have a set price for a given compound where the manufacturer intended to launch the drug, and the manufacturer and the payor would have had a chance to reach a mutual agreement.

Overall, 27 medicines were identified and included in the analysis, which utilized current list prices for the 37 countries in scope that were valid in April 2020. Official price sources from the countries were used via Akceso’s ‘Verity Prices and Reimbursements Tool’ to collect published list prices in local currency; for the United States, the Federal Supply Schedule prices were used. Five brands that had published list prices in less than 20% of the in-scope countries (<=7) were excluded, leaving 22 compounds for further analysis (Table 1). Parallel imports in all countries were excluded from the dataset, as the focus was on the pricing impact of ERP, net of arbitrage effects. Prices were normalized to the ex-factory level using standard margins as specified in each country. For purposes of comparison, all prices were converted to USD, applying 3-months average European Central Bank exchange rates as of April 14, 2020. Price comparisons were made in terms of defined daily dose (DDD) based on the International Non-proprietary Name (INN). If the DDD was not available, we used the daily dose per the label. We calculated the days of therapy (DOT) and ex-factory price per DDD or daily dose (if that made sense according to the label). Within each brand, the Stock Keeping Unit (SKU) identifier was used to determine modal strength and form in a given country. We calculated a country-specific average price per DDD per brand. Given the average price per brand in each country, we calculated the average price worldwide per DDD. To normalize the data, calculated the price ratio for the country for the given brand: the country-specific price was divided by the “world-wide” (ww) average price. Finally, for each country, the average of each product-price ratio was computed, to arrive at the average price ratio of all available brands reviewed, across all the countries.

**TABLE 1:** List of the compounds used in the Price Corridor analysis.

1	Akynzeo^®^
2	Cerdelga^®^
3	Cosentyx^®^
4	Exviera^®^
5	Gardasil^®^ 9
6	Ikervis^®^
7	Jinarc^®^
8	Kengrexal^®^
9	Lenvima^®^
10	Lixiana^®^
11	Mysimba^®^
12	Ofev^®^
13	Opdivo^®^
14	Otezla^®^
15	Quinsair^®^
16	Saxenda^®^
17	Sivextro^®^
18	Synjardy^®^
19	Viekirax^®^
20	Xadago^®^
21	Xydalba^®^
22	Zykadia^®^

### 2.3 Impact on Patient Access

Several analyses were performed to assess the impact on patient access, measured in terms of several indicators.

#### 2.3.1 Impact of Country Income on Access

To assess the impact of country income on access, the availability of medicines in higher-income countries was compared with that in lower-income countries. As explained above, 22 identified medicines, in the thirty European countries were all included in this analysis. The top tertile of countries in terms of average GDP/capita (average: $62,893) was compared with the lowest tertile (average: $14,937). Having an officially published price can be considered as a necessary but not sufficient condition for patient access to a medicine. But when the price of a medicine is not listed in the official price sources for a country, this almost certainly means the medicine is not accessible to patients as per its registered label. The lower- and higher-income countries were compared in terms of the median number of drugs with a listed price from amongst the 22 identified drugs.

#### 2.3.2 Impact of Access Limitations on Patient Health Outcomes: A Case Study of Sacubitril-Valsartan (Entresto^®^, Novartis) for Congestive Heart Failure (CHF)

This case study examines variations in the utilization of an effective, innovative medicine to project the likely reduction in QALYs gained due to access limitations and presumed under-utilization.

##### 2.3.2.1 Selecting a Medicine for a Case-Study Analysis

For a case study of the impact of ERP on access and population health outcomes, we identified a target compound for study from among the innovative medicines launched over the past 5 years. We selected the case study compound based on four criteria: 1) having a similar prevalence for the indication across the countries; 2) addressing a condition with a large patient population (rather than an orphan condition); 3) being available through retail pharmacies thereby providing better quality data on the volume of use; 4) being available in most countries in our study sample; and 5) being seen as an innovative and unique product.

Innovativeness was an important but challenging criterion to implement. For this rating we relied on the French ASMR (Amelioration du Service Medical Rendu, or improvement of medical benefit) rating system. Our review of the French data for 2015–19 found that (excluding indication extensions) 49 assessments had an ASMR rating of 1, 2, or 3 (on a scale from “1” meaning major improvement and to “5” meaning no improvement). The large majority of these were for hospital treatments, some were vaccines, and yet others were orphan drugs. Finally, we found that none of the ASMR 1-2-3-rated compounds were appropriate for our analysis. Among ASMR 4-rated compounds (i.e., those with “minor improvement”), we were able to identify one—sacubitril-valsartan for symptomatic CHF—that fully satisfied our set of criteria. It was a first-in-class retail product that is available in most countries. In addition, the prevalence of CHF is relatively similar across the 34 countries of interest where reliable usage and price data were available.

Two literature searches were conducted to retrieve the prevalence data for CHF as well as the cost-utility modeling studies that could provide an estimate of the projected QALY gain from sacubitril-valsartan treatment.

##### 2.3.2.2 Estimating the Country-specific Prevalence of CHF

The objective was to retrieve prevalence data from the literature for CHF in each country of interest.

The estimates ranged from 1.2% in Belgium to 3.96% in Germany with most prevalence data being estimated based on the general consensus that known heart failure ranges between 1–2% in the developed countries (Störk et al., 2017; Smeets et al., 2019; Groenewegen et al., 2020). Instead of attempting to calculate an average estimate, we decided to rely on the estimate of one country: in this case, Sweden was chosen while others were not for the following reasons: 1) the methodology used to derive epidemiologic data was not of equal quality across studies; 2) some studies used non-standardized criteria while others used standardized criteria; 3) the studies covered different time periods between 2010 and 2019, which could lead to an additional bias when interpreting the findings; and 4) Sweden, on the other hand, had conducted several epidemiological studies on CHF using a rigorous methodology in the last decade. We therefore decided to use the Swedish prevalence estimate of 2.2% reported by Zarrinkoub et al. (2013) (Zarrinkoub et al., 2013).

##### 2.3.2.3 Estimating QALY Impact

A systematic literature research was conducted to retrieve cost-utility studies assessing the value of sacubitril-valsartan in patients with heart failure to collect the present value of projected QALY gains across the studies. The search was conducted in PubMed and Cochrane libraries using the keywords “sacubitril-valsartan,” “QALY”, and “Entresto.” Twenty-eight articles were retrieved, of which were 13 excluded. Reasons for exclusion included 1) the study drug was not sacubitril-valsartan, 2) the condition studied was not CHF, 3) the study did not report QALYs, 4) the comparator to sacubitril-valsartan was not an angiotensin-converting-enzyme inhibitor (Ekman et al., 2008; Pradelli et al., 2009; Taylor et al., 2009; Maniadakis et al., 2011; Baker et al., 2012; García Ruiz et al., 2013; Wu et al., 2013; Stafylas et al., 2015; Chan et al., 2016; Zacà, 2018; Perera et al., 2019; Smith et al., 2019; Ren et al., 2020). The remaining 15 included studies provided 14 cost-utility analyses, and one systematic literature review of CUAs which was used to validate results from the overall search (Gaziano et al., 2016; King et al., 2016; Sandhu et al., 2016; Ademi et al., 2017; Ramos et al., 2017; van der Pol et al., 2017; Gandjour and Ostwald, 2018; Krittayaphong and Permsuwan, 2018; Liang et al., 2018; McMurray et al., 2018; Zueger et al., 2018; Park et al., 2019; Borges et al., 2020; Chin et al., 2020; Liu et al., 2020). The estimated QALY gains for sacubitril-valsartan ranged from 0.29 to 0.79 QALYs (Table 2). Given that a lifetime perspective is preferred in economic analysis for a chronic condition, we excluded studies that considered a time horizon of less than 20 years. Considering only the studies with at least 20 years’ time horizon, different discount rates were used in the individual studies, of 3 and 5%. On average, the studies using 3% showed a QALY gain of 0.66 while those using 5% produced a QALY gain of 0.44. Given that the number of studies was too small for a meaningful regression analysis, we used a simple average of the QALY gain in the selected studies in our model. This resulting average incremental QALYs in present value from the included studies was 0.536. In addition, we conducted deterministic sensitivity analyses for 0.44 QALY gain from studies of 5% discount rate and 0.66 QALY gain from studies with 3% discount rate.

**TABLE 2:** List of published sacubitril-valsartan studies reporting QALY gains.

Authors	Year	Manufacturer funded study	Type of publication	Perspective	Time horizon	Discount rate (benefits)	Source for utilities	QALYs gained
Liu et al	2020		Review					
Borges et al	2020	N	CUA	Societal	30 years	5	PARADIGM-HF trial	0.44
Park et al	2019	Y	CUA	Health care sector	Lifetime	5	General South Korean population	0.59
Chin et al	2019	N	CUA	Health care sector	20 years	5	King et al., 2016	0.31
Gandjour et al	2018	Y	CUA	SHI	Lifetime	3	PARADIGM-HF trial	0.76
Krittayaphong et al	2018	N	CUA	Health care sector	Lifetime	3	Gaziano et al., 2016 King et al., 2016	0.79
Zueger et al	2018	?	CUA	Payor	5 years	3	Griffiths et al., 2014	0.102
McMurray et al	2018	Y	CUA	Health care sector	Lifetime	3.5	PARADIGM-HF trial	0.52
McMurray et al	2018	Y	CUA	Health care sector	Lifetime	3	PARADIGM-HF trial	0.47
McMurray et al	2018	Y	CUA	Health care sector	Lifetime	5	PARADIGM-HF trial	0.42
Liang et al	2018	N	CUA	Health care sector	10 years	3	CARE HF	0.21
Ademi et al	2017	Y	CUA	Health care sector	Lifetime	3	PARADIGM-HF trial	0.4254
Ramos et al	2017	Y	CUA	Societal	Lifetime	1.5	PARADIGM-HF trial	0.33
van der Pol et al	2017	N	CUA	Health care sector	30 years	1.5	SHIFT Study (ivabradine)	0.29
Sandhu et al	2016	N	CUA	Societal	Lifetime	3	PARADIGM-HF trial	0.62
Gaziano et al	2016	Y	CUA	Payor	30 years	3	PARADIGM-HF trial	0.78
King et al	2016	N	CUA	Payor	Lifetime	3	CARE HF	0.76
								
Mean among subset studies (in bold, used for the analysis)					>20 years			**0.5361**

To estimate the 1) treated and 2) eligible treatable (but not treated) patient volumes, we used 2019 utilization data from IQVIA.

##### 2.3.2.4 Patients Treated With Sacubitril-Valsartan

The number of sacubitril-valsartan treated patients was estimated for each country in scope. Population size for each country was collected using 2019 Eurostat database for the European and World Bank database for the non-European countries. We then estimated the number of actual sacubitril-valsartan treated patients per 100,000 inhabitants for each country.

##### 2.3.2.5 Patients Eligible for Sacubitril-Valsartan

While the general prevalence of this condition would suggest a certain number of eligible patients for treatment in each country, this may overstate the practically feasible number of patients treatable by a given drug. Therefore, we identified the country with the highest share of patients treated with sacubitril-valsartan as the baseline for all other countries. This showed Germany providing the highest level of access to the drug—with 132.6 patients treated per 100,000 inhabitants. As a feasibility check on this, we found it to correspond to about 12% of the eligible, prevalent CHF patients in Germany with reduced ejection fraction, i.e., the registered indication for sacubitril-valsartan. Being at only 12% of all eligible patients, our maximum baseline can be therefore considered a conservative benchmark for the share of treatable patients. This benchmark of 132.6 patients per 100,000 inhabitants was used in the next steps of the analysis. Population size for each country was then used to calculate the number of treatable patients per country by multiplying it with the German benchmark. Finally, in each country, the actual number of treated patients vs the treatable number were compared to estimate the number of patients who were treatable but not treated, up to the level of the German access level benchmark.

##### 2.3.2.6 QALYs Gained in Treated and Lost in Untreated Patients

For each country, the number of treated patients was multiplied by the present value of incremental QALY, and these were aggregated to a total for all countries, representing the cumulative actual health benefit gain from treatment in our 34 countries. Similarly, for each country, the number of untreated but eligible patients was multiplied by the QALY gain expected should each receive sacubitril-valsartan treatment. Given volume data for the 34 countries, we could estimate the worldwide QALYs lost due to not treating all eligible patients to the benchmark level.

### 2.4 Impact on Innovation

Assessing the empirical impact of revenues on pharmaceutical R&D is obviously challenging given the lack of a well-researched basis. There are only a few studies that have tried to estimate the “revenue elasticity of innovation”. In addition, this is further complicated by the fact that innovative pharmaceuticals are global goods, whose embedded information has global public good properties. In his comprehensive literature review, Lakdawalla (2018) (Lakdawalla, 2018) concludes: “The preponderance of evidence suggests that raising reimbursements for pharmaceuticals stimulates innovation.” He cites Acemoglu and Linn’s (2004) (Acemoglu and Linn, 2004) estimate that a 1% increase in expected market size being associated with a 4—6% increase in the number of new molecular entities (NMEs) entering the market. Similarly, Dubois et al. (2015) (Dubois et al., 2015) estimate a point elasticity of 0.23 and conclude that the typical required revenue to produce an additional NME is $2.5 billion, while referring also to a range of $1.6 to $2 billion inferred from accounting calculations. These are in the plausible range with DiMasi et al.’s (2016) (DiMasi et al., 2016) estimate of the average development cost of an NME to be $2.6 billion.

Our case study analysis of sacubitril-valsartan can be used to quantify how much additional revenue would be generated for the manufacturer if all patients were treated to the benchmark level, which additional revenue could then be used partially to finance further R&D.

We estimated the average annual treatment cost for each country (based on the per-label recommended dose of two tablets per day at the ex-factory public net price level at 1 July 2020 prices). This was then multiplied by the number of untreated patients eligible for sacubitril-valsartan in each country up to the German access level as the target benchmark. Aggregating these results across countries to estimate additional potential revenues for the manufacturer, and comparing it to the range of $1.6 bn—$2.6 bn per NME provides a high-level estimate of how many additional innovative compounds could be developed given this additional revenue.

## 3 Results

**FIGURE 1:**
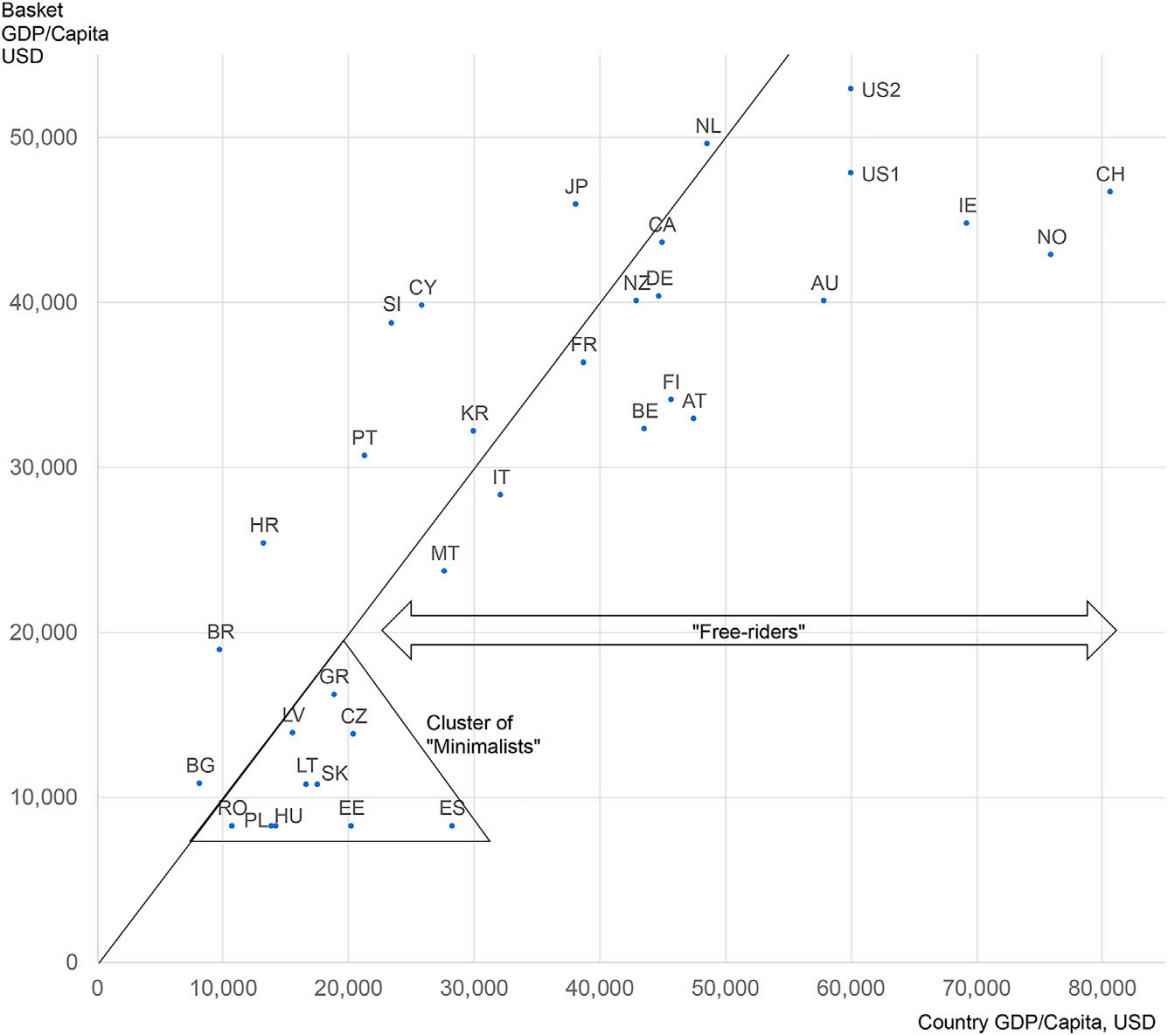
ERP affordability (mis)match map—how far is each country setting its prices based on a country basket of similar affordability Notes: United States data are two hypothetical counterfactuals, based on recent government proposals: United States1 calculated as per “Ways and Means” (2019) (Ways and Means Committee, 2019); United States2 calculated as the maximum of “CBO” (2021) (Congressional Budget Offi, 2021); Canada calculated with rules planned to be valid as of 2022.

One indicator of the potentially distorting effects of ERP is the extent to which a country is setting its prices in relation to a set of countries having different economic situation in terms of ability to pay. Figure 1 depicts these relationships by comparing the GDP per capita of a country with the “basket GDP/capita” calculated by applying the country’s ERP algorithm on the basket of countries it is referencing. Countries on—or very close to—the diagonal line, are ones that determine their prices based on the prices of countries of similar income level. Numerous wealthy countries—to the right side of the diagonal line—are, however, attempting to “free-ride” by referencing the prices of lower-income countries. Countries to the left side of the diagonal are benchmarking their prices to countries with a higher income level, thus potentially “shooting themselves in the foot” by importing higher prices. And finally, at the lower end of the line, we find a “Cluster of Minimalists”, i.e., lower income countries that apply a price-minimizing algorithm to their basket seeking even lower prices: countries in this cluster are almost always in the free-rider category. Since the United States does not currently use ERP, the United States indicator is only hypothetical, reflecting the likely impact of proposals under consideration.

**FIGURE 2:**
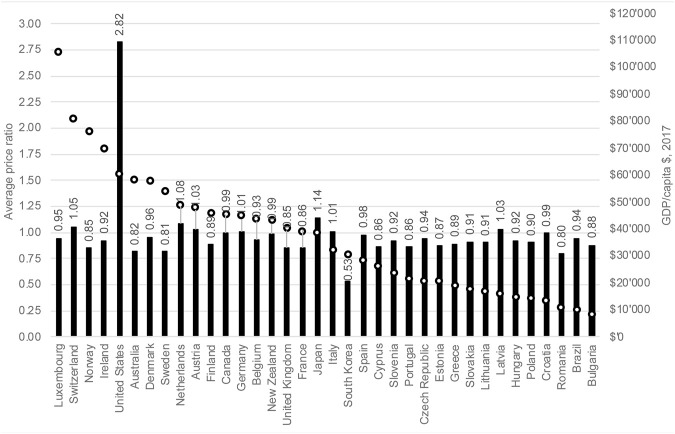
Relative launch product prices vs affordability.

But what is the impact of these ERP referencing patterns on actual price differentials? Figure 2 summarizes the differences based on the 22 compounds in our 2015 comparison set. Among the countries other than the United States there is no clear correlation between the pharmaceutical price level and the income level of the country. Average pharmaceutical prices are largely independent of GDP/capita: this is quite similar to the pattern shown in Danzon (2018) (Danzon, 2018). In fact, the price corridor of countries is relatively tight, with two noticeable outliers—the United States, which did not apply ERP at the time of the analysis, as an upward outlier and South Korea as a downward outlier. This analysis is generally based on list prices: a net price comparison, which is not feasible to produce due to the confidentiality of rebates and net prices in most countries, would show the United States as less of an outlier, however.

**FIGURE 3:**
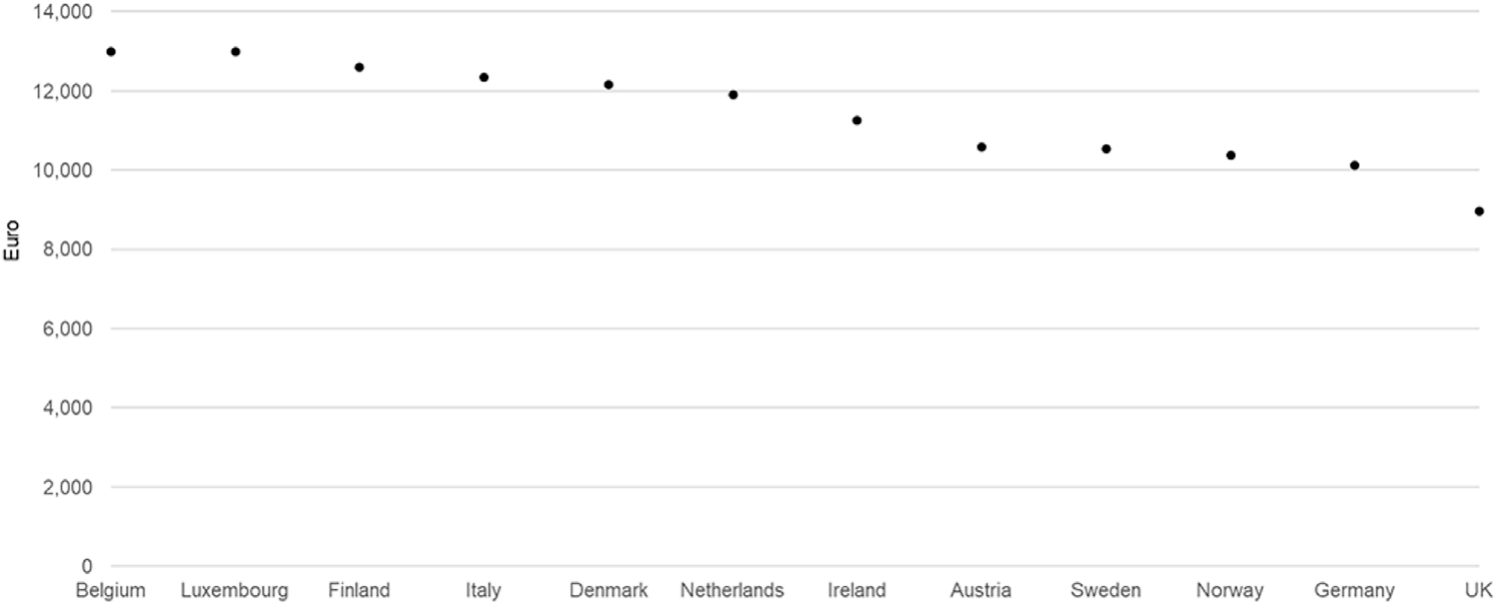
Lumacaftor/ivacaftor ex-factory prices in all European countries where listed (200mg/125mg, 112x, November 2019 data).

One possible explanation for the tight price corridor independent of the ability to pay among countries using ERP is avoidance strategies driven by the manufacturers’ business objectives to avoid ERP-driven price erosion—the so-called “race to the bottom”. Avoidance strategies result in many new drugs being unavailable to patients in lower ability-to-pay countries due to no launch or extended negotiations, thereby allowing manufacturers to avoid the ERP spillover effect to other, higher-income markets with greater profit potential. For example, lumacaftor/ivacaftor for treating cystic fibrosis had a relatively tight pricing corridor while having listed ex-factory prices only in 12 higher-income European countries 4 years after marketing authorization by EMA. It was not listed in any of the lower-income ones, demonstrating an ‘Avoidance’ strategy (Figure 3).

Our access analysis of availability showed that—of the 22 new drugs included in our purposive sample—five years after registration, a median number of 11—or only about 50%—was available in the lower-income countries. In the higher-income countries, this median availability was 18 drugs, or 82%.

Importantly, practically no large country—with a large healthcare market—is among the lower-income ones. Thus, manufacturers are delaying or even avoiding launch in the smaller market-potential countries in order to not jeopardize the revenues and margins of their large markets. And, presumably, even if they do start price negotiations, they are not reaching an agreement due to the mismatch between the ability to pay of the country and the expected price level by the manufacturer. Manufacturers would often be willing to accept lower prices—as shown by, for example, using tender offers that with a few exceptions do not constitute ERP risk—and thereby match the ability to pay of lower-income countries. However, with the prevailing risk of their large markets using national prices for referencing even if in theory they could be confidential net prices, deters them from doing so. Furthermore, recent, newly invigorated demands of certain large countries for price transparency can prompt an even stronger defensive reaction from companies. All of these lead to little flexibility for both list and net prices, further limiting patient access especially in lower-income countries that have lower market potential.

Even if a product is “available” in theory in a country—i.e., has a published, reimbursed price—it normally faces covert access barriers. These are indirectly due to ERP encouraging avoidance strategies that do not support affordable pricing in lower-income countries. This is illustrated in our case study of patient access to sacubitril-valsartan, comparing utilization to a German benchmark. For the 2019 cohort of patients in 34 advanced countries we project a potential aggregate health gain (in terms of discounted lifetime QALYs) of 335,000 for CHF patients receiving sacubitril-valsartan treatment. On the other side, many eligible patients don’t receive this innovative treatment for CHF: taking the German level of access to the medicine as a baseline—which could be considered a conservative estimate of the potential use of this effective treatment—the projected foregone aggregate QALY gain in the other 33 countries would amount to 507,000 QALYs (range: 416,202–624,302 QALYs) according to the sensitivity analyses for different discount rates. Given the nature of this disease condition, this means not only reductions in quality of life, but clearly many human lives are lost prematurely.

## 4 Discussion

### 4.1 Key Findings

Pharmaceutical companies operating for profit are the drivers in making innovative medicines available to patients by developing, registering, manufacturing, and marketing them. And these medicines can improve the health status of current and future generations worldwide. A recent analysis of life expectancy gains in the United States between 1990 and 2015 attributed 35% of the 3.3 years improvement to pharmaceuticals (Buxbaum et al., 2020). In pursuit of their profit objectives, companies strive to optimize prices, recognizing that the net realized price of a medicine in a country is constrained by its achievable list price as a ceiling. List prices, in turn, are determined in many markets by ERP policy.

Our analysis shows that many large higher-income countries are free-riding by referencing the prices of countries with less ability to pay. These free-riding countries are able to exploit their monopsony power to gain lower prices than their income could support. Also, perversely, in effect, some lower-income countries are subsidizing some higher-income ones which obtain lower prices than they could actually afford. And due to the nonlinear dynamic of the global ERP network, this subjects pharmaceutical manufacturers to the risk of a “race to the bottom” in pricing. Pharmaceutical companies, therefore, try to maintain a tight list price corridor for their launches and throughout the product lifecycle.

They limit the global pricing corridor mainly by two strategies: 1) setting a floor price and 2) “avoidance” or “launch delay” strategies. By setting a floor price that many lower-income countries cannot afford, this may block the treatment for patient populations in these countries who could benefit from it.

ERP can also trigger additional avoidance strategies by pharmaceutical companies, often manifesting themselves in either non-launch in lower-income countries offering lower market potential, or even withdrawal of the products in case of price revisions—e.g., as in the German G-BA system after 1 year. They fear that cost-containment measures targeting pharmaceutical prices in these countries would threaten prices and thus revenues in other countries *via* ERP.

ERP indirectly causes additional covert access limitations by which patients are withheld innovative treatments in lower-income countries and even in some higher-income ones that apparently want to go below the floor price.

As our case study with sacubitril-valsartan illustrates, the resulting health loss can be very substantial. For this one compound alone, we arrived at a conservative estimate of an annual loss of 507,000 QALYs in 34 countries, reflecting many avoidable deaths. For reference, although being a conservative estimate, this is an order of magnitude higher than what Uyl-de Groot et al. (2020) (Uyl-de Groot et al., 2020) found with 30,000 life-years lost due to market access delays for two cancer medications in 28 European countries.

In addition to the lives and QALYs lost by limiting access to effective treatments, the implied reduction in revenues means less future innovation: lower utilization also implies lower revenues for manufacturers and can mean fewer funds for R&D. We estimate that providing the same level of access in all investigated countries as in Germany would mean $2.06 billion additional annual revenue from these countries alone in this case study. This reduction can imply less investment in R&D in the long run at a global level, too.

While the lack of data availability allowed us to model only the case of this one innovative treatment, we can extrapolate to very substantial adverse population health consequences if we consider all the current treatments restricted due to ERP, already with the currently operating worldwide system.

Were the United States to implement ERP, the market dynamic would most probably shift considerably towards even more widespread avoidance strategies. We believe the impact should be examined through three channels of impact. First, there would be an impact on the static efficiency for in-market drugs in the United States. In the short run, these price cuts could result in higher utilization. This could, in parallel, freeze any market access rollout in other countries by manufacturers wary of ripple effects. Second, there could be a short-term impact on static efficiency for drugs about to be launched in the United States with manufacturers offering little or no price concession ex-United States compared to previously planned price levels. Furthermore, the accompanying launch avoidance could be extended to large EU countries, Australia, Japan, Korea, resulting in worse global patient access and declines in pharma revenues. Third, there would be the long-term negative impact on innovation (dynamic inefficiency) as fewer medicines are developed. This is due to having fewer funds for R&D, with the ultimate result being fewer new, innovative treatments available to future patients in the world.

### 4.2 Limitations

For this research we used the published country-specific list prices, or, where available, net prices after official mandatory discounts. While these are the ones being used for actual ERP calculations, for projecting the full dynamic of the access limitations and R&D opportunities, net prices would be preferable. Net prices are, however, confidential in most countries. Although we buttress our general analysis with a single case study, the results are in line with conclusions from the literature and our overall empirical analysis. Of course, the generalizability of our findings could be strengthened by further case studies with a similar analytic approach applied to medicines in other therapeutic areas.

## 5 Conclusion

As implemented today, a WHO pricing guideline concludes that there is a “balance of effects of external reference pricing in favor of implementing the policy” (WHO, 2020), but still this policy has significant unintended consequences—some overt and others less apparent. Clearly, it can limit access to treatments in some markets where manufacturers have less incentive to sell, resulting in worse morbidity and mortality outcomes for today’s patients in those countries. And these revenue reductions—due to higher-income countries demanding the same prices as lower-income countries obtain—will affect future patients by lowering R&D and thus producing less innovation in the long term. Should the United States implement ERP, we anticipate these effects will be magnified, with companies choosing to not launch even in large high-income EU countries to protect their United States prices and hence their global business. The collective result is everyone will be “shooting themselves in the foot” (Incze et al., 2020), reducing pharmaceutical innovation for short-term saving that hurts patients worldwide in the long term. Globally, many patients suffer from health conditions that, without access to effective treatments, could mean they would die prematurely as a result.

## Data Availability

The raw data supporting the conclusion of this article will be made available by the authors, without undue reservation.
